# Triatrial Appearance in a Patient With Atrial Septal Defect: A Case Report

**DOI:** 10.7759/cureus.84011

**Published:** 2025-05-13

**Authors:** Bhushan Shah, Abhimanyu Uppal, Shekhar Kunal, Sudesh Prajapati, Ankit Gupta

**Affiliations:** 1 Cardiology, All India Institute of Medical Sciences, Bhopal, Bhopal, IND; 2 Cardiology, Priyanka Hospital and Cardiac Center, Jaipur, IND; 3 Cardiology, Employees' State Insurance Corporation (ESIC) Medical College and Hospital, Faridabad, IND; 4 Cardiology, All India Institute of Medical Sciences, Raebareli, Raebareli, IND

**Keywords:** atrial septal defect, computed tomography, cor triatriatum, echocardiography, imaging

## Abstract

Atrial septal defect (ASD) is a common congenital heart anomaly, often diagnosed with relative ease. However, the presence of additional structural abnormalities, such as cor triatriatum dexter (CTD) or a prominent eustachian valve, can create a triatrial appearance, complicating diagnosis and management. Accurate differentiation of these anomalies is essential to guide appropriate clinical decisions.

We report the case of a 25-year-old male with exertional dyspnea and fatigue. Cardiovascular examination revealed a wide, fixed splitting of S2 and a systolic murmur. Transthoracic echocardiography (TTE) suggested an ostium secundum ASD (OS-ASD) with an abnormal membrane in the right atrium, raising suspicion for CTD. Further evaluation with transesophageal echocardiography (TEE) and cardiac computed tomography (CT) revealed an incomplete CTD (iCTD) rather than a true triatrial division. Cardiac catheterization confirmed an operable left-to-right shunt with mild pulmonary arterial hypertension. The patient underwent successful ASD closure and membrane excision.

This case highlights the importance of multimodality imaging in distinguishing a triatrial appearance in ASD patients. Misdiagnosis of iCTD as CTD or a prominent eustachian valve can lead to inappropriate management decisions. Advanced imaging techniques, including three-dimensional TEE (3D TEE) and cardiac CT, are crucial for precise anatomical assessment and surgical planning, ensuring optimal patient outcomes.

## Introduction

Atrial septal defects (ASDs) are among the most common congenital heart diseases [1]. While diagnosing ASDs is relatively straightforward, the presence of additional anomalies can complicate clinical evaluation and management. In normal individuals, the atrium is usually divided into right and left atria by the interatrial septum, giving a biatrial appearance. A large membrane in the right atrium (cor triatriatum dexter (CTD)) or a prominent eustachian valve may create the appearance of a triatrium by creating a misleading impression of an extra chamber in the right atrium [2,3]. Incomplete CTD (iCTD), a condition where the right atrium is incompletely divided by the membranous band, should be differentiated from complete CTD using three-dimensional transesophageal echocardiography (3D TEE) and cardiac computed tomography (CT), as they may appear similar on transthoracic echocardiography (TTE). Differentiating between ASD and ASD associated with iCTD or a redundant eustachian valve is critical for determining appropriate management, especially when planning interventions such as transcatheter closure [4-6].

This report describes an interesting case where triatrial appearance in a patient with ASD was due to a fibromuscular membrane in the right atrium. We have focused on distinguishing the different forms of CTD, namely incomplete and complete CTD, through multimodality imaging techniques for precise understanding of these rare anomalies, facilitating accurate diagnosis and guiding appropriate clinical management.

## Case presentation

**Figure 1:**
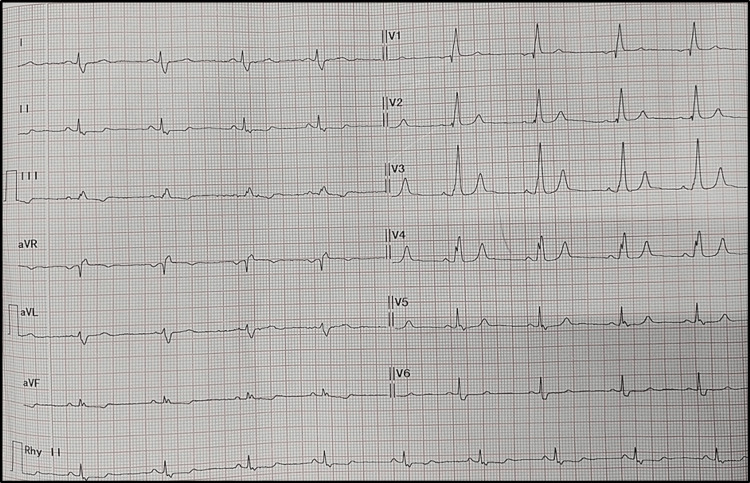
The 12-lead ECG showing normal sinus rhythm (NSR) with right bundle branch block (RBBB). Image Credits: Dr. Bhushan Shah

A 25-year-old male presented with complaints of exertional dyspnea and fatigue for the past 12 months. He had no history of chest pain, palpitations, or syncope. The patient’s vital signs were normal, with peripheral oxygen saturation (SpO2) of 98% on room air. No bluish discoloration or digital clubbing was observed. Cardiovascular examination revealed a normal-shaped precordium with no obvious bulge or pulsation. The apex beat was palpable in the left fifth intercostal space at the midclavicular line. On auscultation, S1 was normal, P2 was loud and wide, and fixed splitting of S2 was noted. A grade II ejection systolic murmur was heard in the left second intercostal space. No S3, S4, or other added sounds were present. He was evaluated on an outpatient basis with an electrocardiogram (ECG), TTE, and chest x-ray. The ECG revealed a normal sinus rhythm with right bundle branch block (Figure 1).

**Figure 2:**
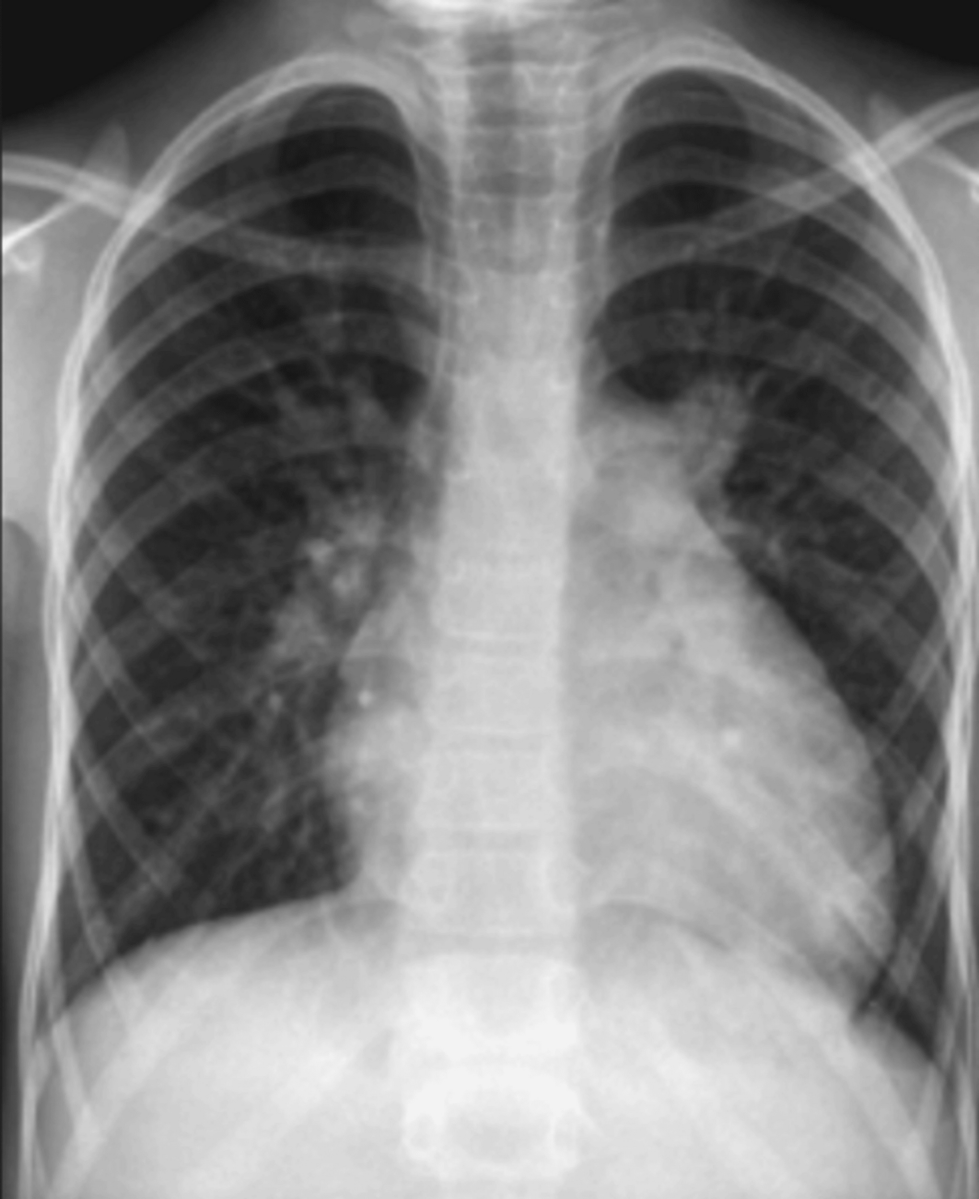
The posterior-anterior chest x-ray of the patient showing dilated right atrium with prominent pulmonary arteries.

**Figure 3:**
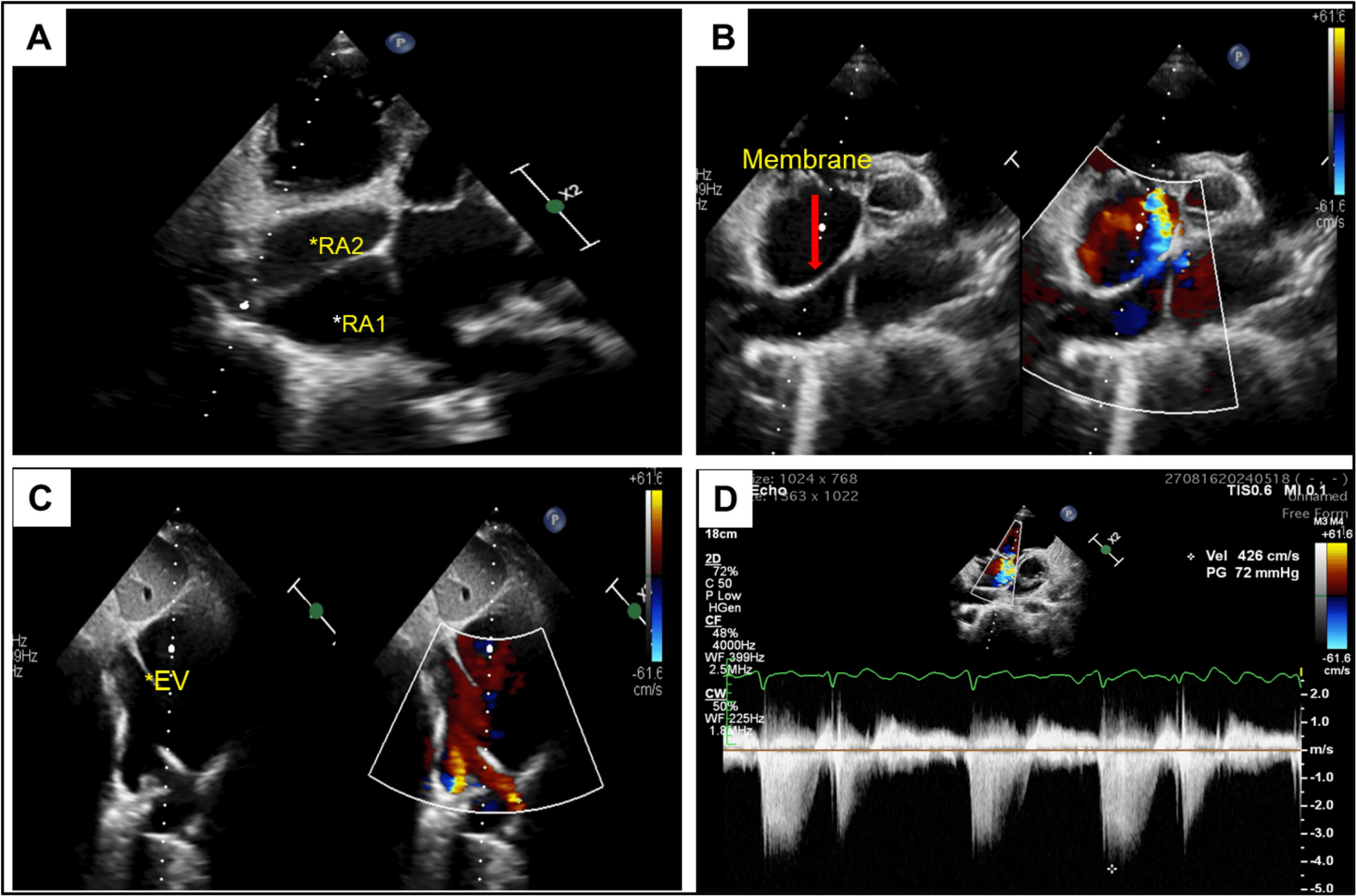
Transthoracic echocardiographic images. (A) Modified off-axis apical four-chamber view showing a membrane dividing the right atrium into two chambers right atrium 1 (RA1) and right atrium (RA2) giving triatrial appearance. (B) Parasternal short-axis view showing the interatrial septum and a tissue band arising posteriorly, attaching to the interatrial septum near the aortic end. (C) Subcostal bicaval view showing a prominent eustachian valve (marked as EV with an asterisk), atrial septal defect with left-to-right shunt. (D) Continuous wave Doppler across tricuspid valve showing severe tricuspid regurgitation and severe pulmonary arterial hypertension.

**Figure 4:**
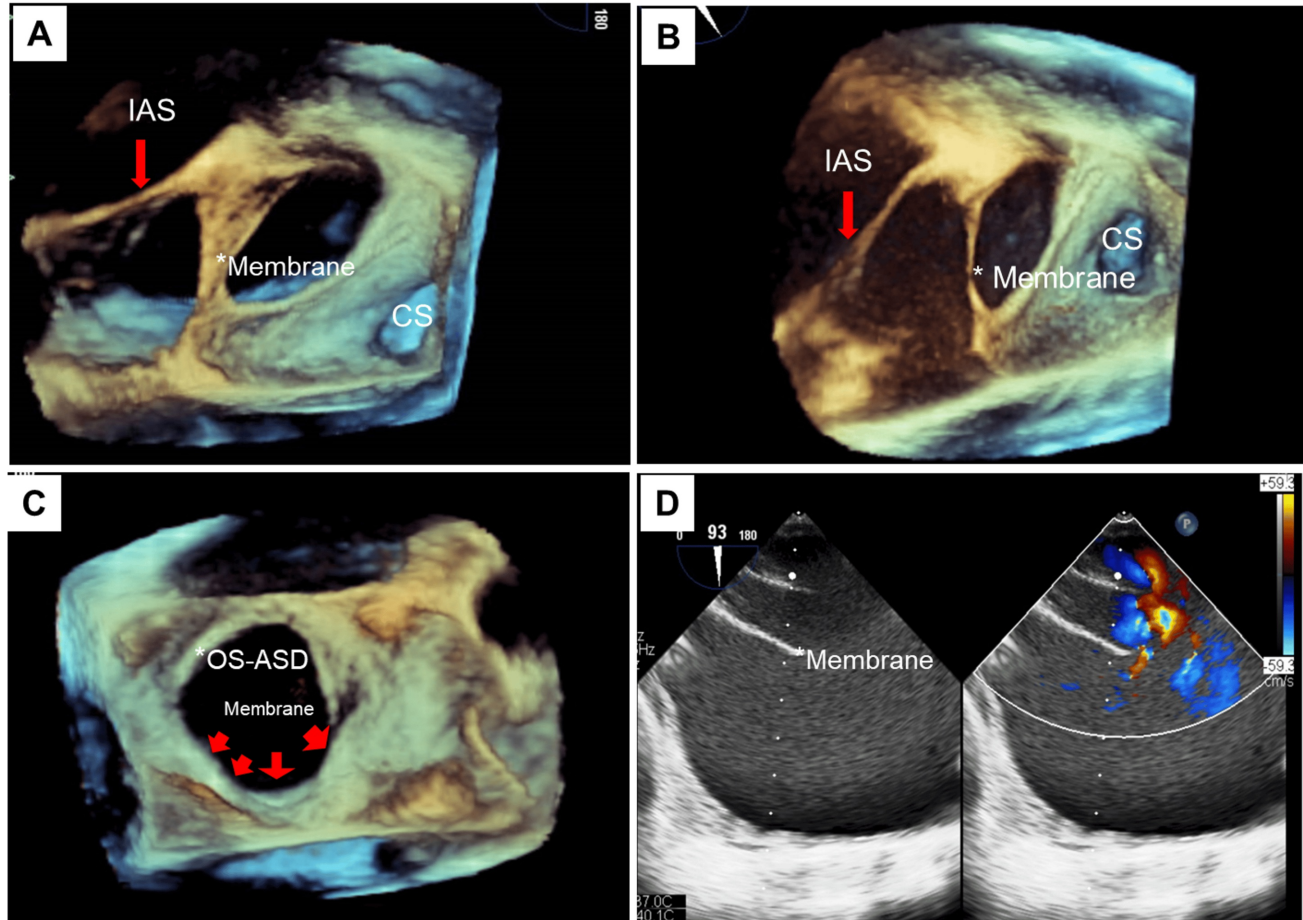
Transesophageal echocardiography images. (A, B) Modified three-dimensional right atrium enface transesophageal echocardiography view images showing interatrial septum (IAS) with ostium secundum atrial septal defect (OS-ASD) with prominent eustachian valve/membrane giving the appearance of a divided right atrium. (C) Three-dimensional transesophageal echocardiography right atrium enface view showing a large OS-ASD. (D) Two-dimensional transesophageal echocardiography bicaval view showing a prominent eustachian valve and the inferior vena cava rim of the atrial septal defect.

**Figure 5:**
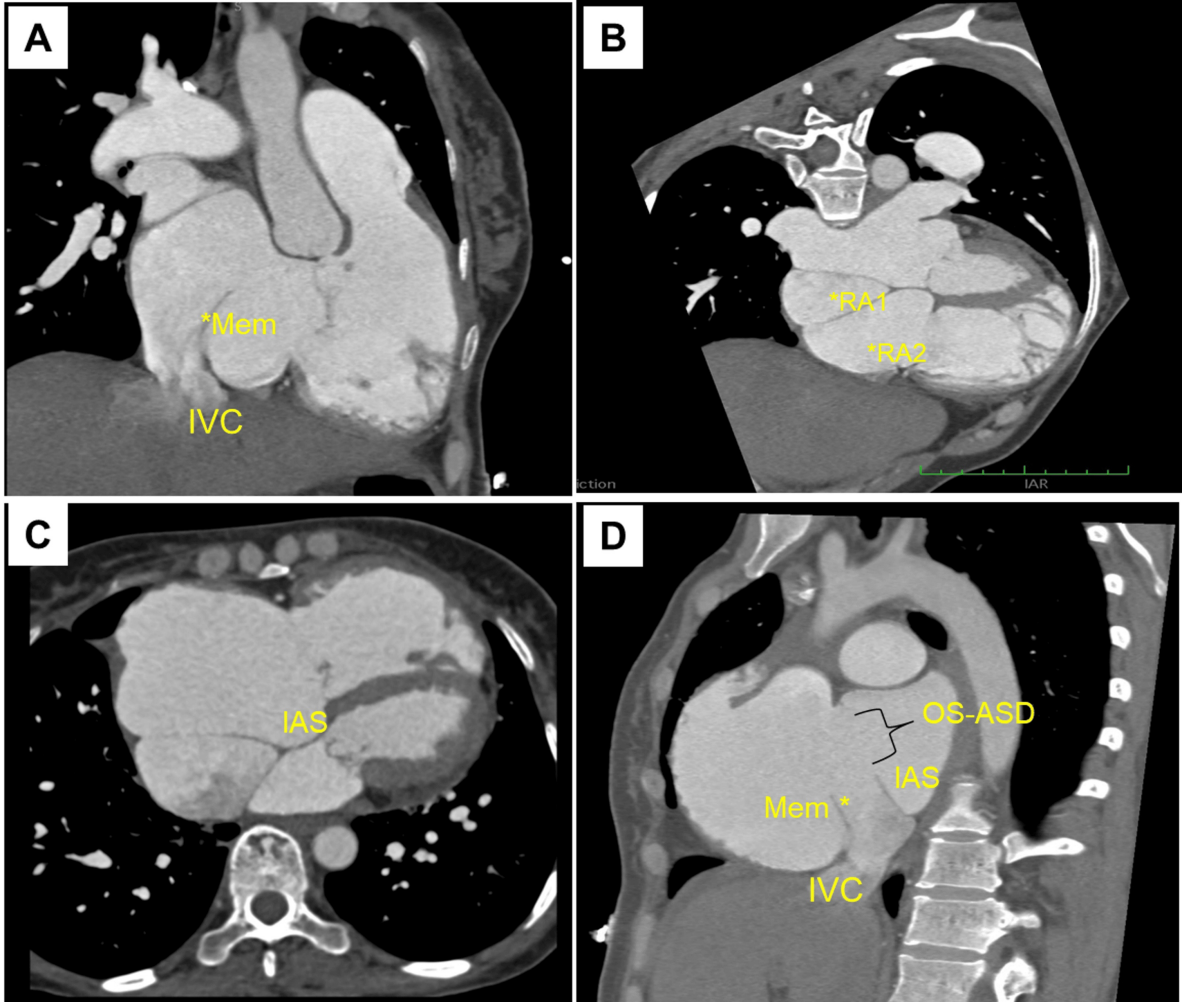
Cardiac computed tomography images. (A) Right ventricle two-chamber view showing a membrane from the anterior rim of inferior vena cava (IVC). (B, C) Four-chamber view showing a divided right atrium (RA1 and RA2) due to a membrane, along with a large ostium secundum atrial septal defect (OS-ASD). (D) Modified biatrial view showing the membrane (mem), IVC, interatrial septum (IAS) and OS-ASD.

The chest x-ray in the posterior-anterior (PA) view showed right atrial enlargement with prominent pulmonary arteries (Figure 2). TTE revealed an abnormal membrane-like structure in the right atrium, dividing it into two chambers. This structure originated from the lateral wall of the right atrium, just above the ostium of the inferior vena cava (IVC), and extended to the middle of the interatrial septum, simulating CTD. The right atrium and right ventricle were dilated, with significant tricuspid regurgitation. They elevated right ventricular systolic pressures (up to 72 mm Hg), suggestive of severe pulmonary arterial hypertension (PAH). The hepatic and IVC were dilated. An ostium secundum ASD (OS-ASD) measuring up to 28 mm with a deficient posterosuperior rim and a left-to-right shunt was noted (Figures 3A-3D). To differentiate between iCTD and complete CTD, 3D-TEE imaging was performed. TEE demonstrated a membrane arising from the anterior border of the IVC, extending to the atrioventricular margin and the retroaortic margin of the ASD (Figures 4A-4D). The bicaval view in TEE specifically demonstrated an island of deficient membrane in the posterosuperior part of the fibromuscular membrane, raising suspicion of iCTD (Figure 4D). A cardiac CT was done to further delineate the anatomy of the fibromuscular septum. Cardiac CT images confirmed that the fibromuscular band dividing the right atrium into two distinct chambers was incomplete, and the fibromuscular septum was deficient in many aspects, confirming iCTD (Figures 5A-5D).

**Figure 6:**
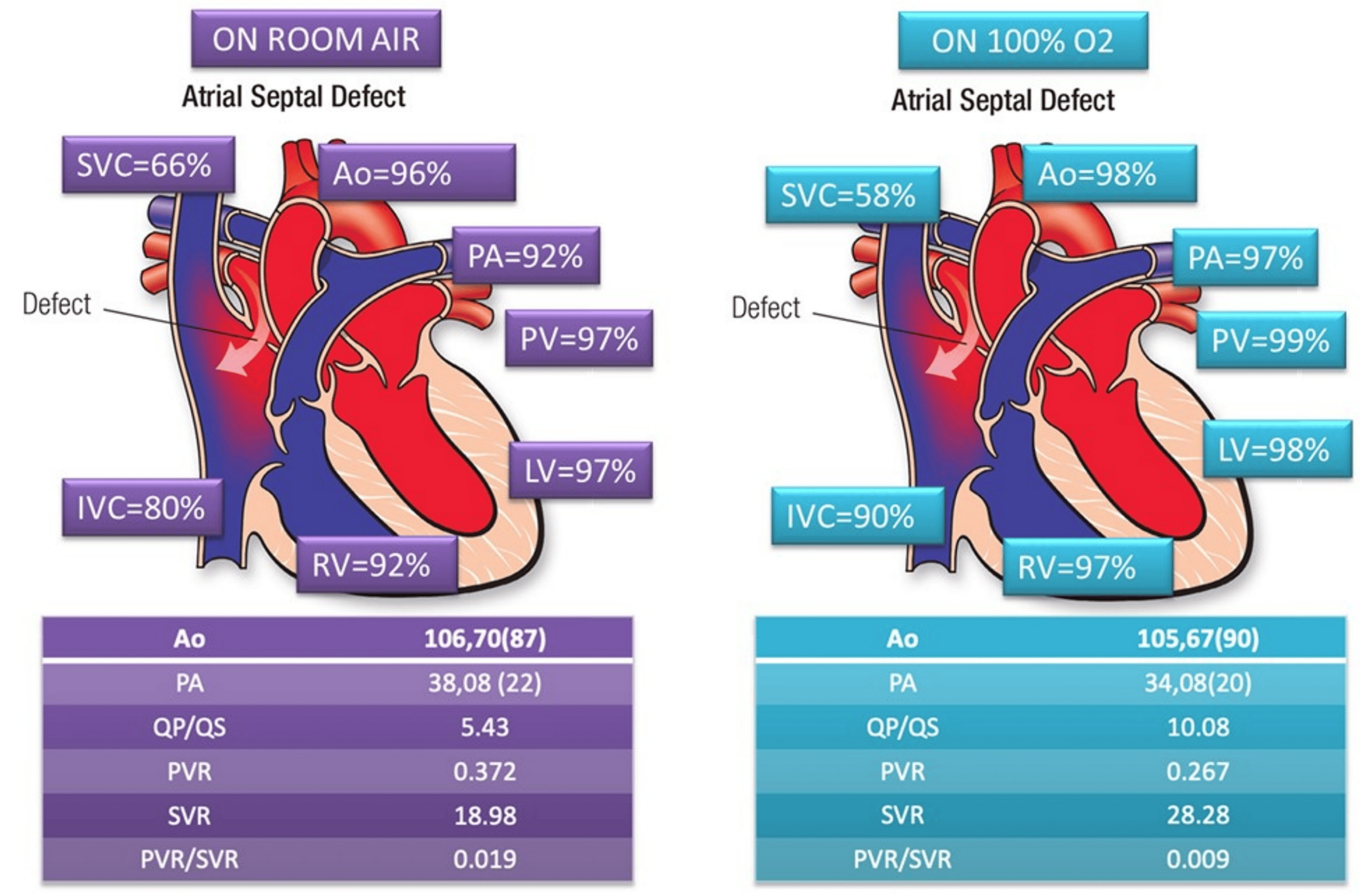
Hemodynamic parameters obtained during cardiac catheterization at baseline, after giving 100% oxygen. SVC: superior vena cava; Ao: aorta; PA: pulmonary artery; PV: pulmonary vein; LV: left ventricle; RV: right ventricle; IVC: inferior vena cava; QP/QS: pulmonary-to-systemic blood flow ratio; PVR: pulmonary vascular resistance; SVR: systemic vascular resistance; PVR/SVR: ratio of pulmonary vascular resistance (PVR) to systemic vascular resistance (SVR); O2: oxygen. Image Credits: Dr. Bhushan Shah

The patient underwent cardiac catheterization with vasoreactivity testing using high-flow oxygen (10 L/min via a mask). Oximetry analysis showed normal systemic arterial and venous saturation. A significant step-up in oxygen saturation at the level of the right atrium indicated an ASD with a left-to-right shunt. Pressure data revealed marginally elevated right atrium pressures, normal left ventricular (LV) and aortic pressures without any gradient between LV and aorta and elevated right ventricular systolic pressures and pulmonary arterial pressures, consistent with mild PAH (Figure 6). No significant gradient was noted within the right atrium across the membrane, suggesting that it was an incidental finding and the anomaly did not have any hemodynamic significance. The pulmonary vascular resistance/systemic vascular resistance ratio in both pre- and post-oxygenation samples indicated an operable shunt. The patient had a deficient posterosuperior rim, so surgical closure of the ASD and excision of the membrane were done.

## Discussion

During fetal development, the right sinus venosus valve plays a crucial role in directing oxygenated blood from the IVC away from the tricuspid valve and through the foramen ovale into the left atrium (LA). Normally, this valve regresses after birth, forming the smaller eustachian and thebesian valves. However, incomplete regression can result in a spectrum of persistent structures within the right atrium, including a prominent eustachian valve, the Chiari network, or CTD [4]. These remnants can create a challenging “triatrial appearance.” Accurate differentiation is essential for diagnosis and management planning.

The Chiari network is a remnant of the incomplete resorption of the right sinus venosus valve, extending across the posterior wall of the right atrium as a reticular, curvilinear structure within the right atrial cavity. This mobile, fenestrated mesh can serve as a potential nidus for thrombus formation and may contribute to pulmonary embolism.

A prominent eustachian valve occurs when the right sinus venosus valve has partially regressed. It is a thin ridge or a crescent-shaped fold of endocardium that arises from the anterior rim of the IVC, protrudes several centimeters into the right atrial cavity, and lacks septal attachments or the appearance of a divided atrium.

Even when the eustachian valve persists, most patients remain asymptomatic and do not require treatment. However, there have been case reports suggesting that prominent eustachian valves, in conjunction with ASD, may contribute to paradoxical embolism or platypnea-orthodeoxia syndrome [7]. A prominent eustachian valve extending into the lower portion of the atrial septum could be misidentified as the inferior rim of an ASD during device or surgical closure, potentially leading to significant iatrogenic right-to-left shunting and worsened post-procedure hypoxemia [6]. This underscores the importance of using TEE and cardiac CT for precise anatomical assessment, identification of eustachian valve variations, and effective management of complications arising from misinterpretation (Table 1). Our report reinforces and expands upon previous literature by emphasizing the diagnostic challenges in differentiating a large eustachian valve from CTD, a rare congenital anomaly with an incidence of approximately 0.025% [3].

**Table 1:** Differentiating prominent eustachian valve, incomplete cor triatriatum dexter, and complete cor triatriatum dexter. ASD: atrial septal defect; IVC: inferior vena cava; SVC: superior vena cava; RA; right atrium

Feature	ASD with prominent eustachian valve [4,6,7]	Incomplete cor triatriatum dexter [4,8]	Complete cor triatriatum dexter [3-5]
Anatomy	ASD with a prominent remnant of the eustachian valve	Divided right atrium by a fibromuscular membrane but incompletely	Divided right atrium by a fibromuscular membrane
Clinical presentation	Often asymptomatic; dyspnea on exertion, possible murmur	Often asymptomatic; dyspnea on exertion, possible murmur	Dyspnea, fatigue, cyanosis, Right heart failure symptoms
Echocardiography	ASD with prominent Eustachian valve; turbulent flow across the defect	A partial membrane visible in right atrium dividing it into two chambers. Membrane extends from the anterior border of the IVC to the atrioventricular border of the ASD, and, in some cases, as far as the retroaortic margin. No to mild obstruction. Transmembrane gradient absent	A membrane visible in right atrium dividing it into two chambers. Upstream chamber getting flow from SVC and IVC while the downstream chamber has RA appendage. Severe obstruction of right atrial flow. Transmembrane gradient present
Management	Surgical or percutaneous closure of ASD	Surgical or percutaneous closure of ASD	Surgical resection of membrane
Prognosis	Good with treatment; risk of iatrogenic right to left shunting during ASD device closure	Good with treatment. High risk of complications during ASD device closure	Excellent with surgical intervention; risk of complications if untreated

CTD is a rare anomaly characterized by a more substantial fibromuscular membrane that typically divides the right atrium into two chambers, often causing significant flow obstruction and a transmembrane gradient. CTD is frequently associated with right-sided anomalies and ASD. An iCTD is a variant where the remnant does not fully divide the right atrium. While it typically results in no to mild obstruction and lacks a significant transmembrane gradient, its presence can complicate interventions [8].

In our case, initial TTE suggested an abnormal membrane dividing the right atrium, simulating CTD, alongside an OS-ASD. However, multimodality imaging, including TEE and cardiac CT, provided detailed anatomical views that clarified the diagnosis as iCTD coexisting with a large OS-ASD. Cardiac catheterization confirmed an operable left-to-right shunt with mild PAH but revealed no significant pressure gradient within the right atrium, supporting the iCTD diagnosis.

Although the iCTD in this patient did not cause significant hemodynamic obstruction, its anatomical relationship to the large ASD was highly relevant to management. Recognizing variants like iCTD is crucial because they may increase the risk of device closure failure and complicate transcatheter procedures. The membrane's proximity to the ASD margins presents practical challenges during device closure, such as interfering with device positioning, seating, or stability, potentially leading to device embolization or a residual shunt. The risk of misidentifying the membrane as part of the septal rim, similar to a prominent eustachian valve, also exists. Such anatomical complexity necessitates customized management approaches.

In view of the complex anatomy involving the large OS-ASD with deficient posterosuperior rim and the coexisting iCTD membrane positioned near the defect margins, which presented a high risk of complications during ASD device closure, surgical closure of the ASD and excision of the membrane were performed. The extent and location of the membrane, coupled with the large defect size, favored the surgical approach to ensure complete and durable closure while simultaneously removing the membrane that posed a risk during percutaneous intervention. In this context, membrane resection was essential as part of the surgical strategy chosen due to the challenging anatomy for device closure, rather than being incidental.

This case highlights the importance of multimodality imaging, including 3D TEE, contrast echocardiography, and cardiac CT for proper identification and differentiation of right atrial membrane-like structures, particularly in the context of interventional planning. The patient recovered well post-procedure, with symptom resolution and no residual shunt on follow-up. This report contributes additional clinical and imaging insights that expand the understanding of this spectrum of congenital anomalies and reinforce the diagnostic and interventional challenges involved.

## Conclusions

We report a diagnostic challenge where a triatrial appearance on TTE, in a patient presenting with exertional dyspnea and fatigue, along with an associated ASD, initially led to suspicion of CTD. Accurate differentiation of such anomalies is essential for guiding appropriate clinical decisions. Advanced imaging techniques clarified the anatomy, confirming an iCTD coexisting with a large OS-ASD.

This case emphasizes the critical role of multimodal imaging in evaluating complex right atrial anatomy. TEE, particularly 3D TEE, offers detailed visualization of intracardiac structures, while cardiac CT provides precise anatomical mapping essential for interventional planning. Although cardiac MRI was not used here, it can be valuable in select cases requiring functional assessment. Recognizing variants like iCTD is essential, as their anatomical relationship to septal defects may significantly increase the risk of device closure failure and necessitate customized management approaches. The presence of the iCTD membrane near the ASD margins presented a high risk of complications during ASD device closure, such as interfering with device positioning or stability. Given the challenging anatomy for percutaneous transcatheter intervention, surgical closure of the ASD and excision of the membrane were performed in this patient. The surgical approach was chosen to ensure complete and durable closure while simultaneously removing the membrane posing a risk during device intervention. The patient recovered well post-procedure, with symptom resolution and no residual shunt on follow-up, reinforcing the clinical relevance of the accurate diagnostic process and tailored management.
